# Genome Sequence of Herpes Simplex Virus 1 Strain SC16

**DOI:** 10.1128/genomeA.01392-16

**Published:** 2017-01-26

**Authors:** Alberto Rastrojo, Alberto Domingo López-Muñoz, Antonio Alcamí

**Affiliations:** Department of Virology and Microbiology, Centro de Biología Molecular Severo Ochoa (Consejo Superior de Investigaciones Científicas and Universidad Autónoma de Madrid), Cantoblanco, Madrid, Spain

## Abstract

Herpes simplex virus 1 (HSV-1), also known as *Human herpesvirus 1*, is a highly prevalent human neurotropic pathogen that causes a variety of diseases, including lethal encephalitis. Here, we report the genome sequence of the HSV-1 strain SC16.

## GENOME ANNOUNCEMENT

Herpes simplex virus 1 (HSV-1) is a member of the *Alphaherpesvirinae* subfamily. HSV-1 establishes latency in the sensory ganglia of the peripheral nervous system, affecting around 90% of the adult population of industrialized countries (1). HSV-1 strain SC16 (2) is one of the most frequently used strains in research, and therefore, the availability of a reference genome sequence is a valuable resource for future studies.

For viral DNA purification, infected Vero cells were harvested 48 h postinfection, subjected to three freeze-thaw cycles, and cellular debris was removed by low-speed centrifugation. Viral particles were concentrated by two sucrose cushions and treated with DNase I to eliminate cellular DNA. Viral particles were lysed using the proteinase K-SDS method, and DNA was purified using the phenol-chloroform method. The NEBNext kit was used for library preparation, and sequencing was carried out in a MiSeq machine at the Parque Científico de Madrid, Spain, obtaining 923,020 paired-end reads (2 × 250 bp).

Contaminating reads were eliminated by aligning reads against *Chlorocebus sabaeus*, the closest relative of Vero cells in the database, and the PhiX174 genome using Bowtie 2 (3). Paired reads were separately aligned to remove all pairs with at least one of the mates mapped to contaminating genomes. Quality filtering was carried out using Prinseq (4). Only paired reads were used for *de novo* assembly of the genome using SPAdes (5), producing 5 contigs (>1 kb) with coverages ranging from 230× to 790×. Then, reads were aligned against contigs using Bowtie 2 (87.65% aligned reads) to remove contig ends with coverage lower than 10×.

Terminal repeat long (TRL) and internal repeat long (IRL) regions were assembled into two contigs with 5,441 and 998 bp in length. The unique long (UL) region was assembled into two overlapping contigs (92,266 and 15,650 bp) and joined using Minimus2 (6). Two major differences against the HSV-1 strain 17 genome (7) were a gap of 185 nucleotides (nt) in a tandemly repeated region, within the UL36 gene locus, which reduced the length of a proline-glutamine repeated motif, and the absence of the OriL sequence, a perfect palindromic sequence required for viral DNA replication (8). The alignment of the reads against HSV-1 strain 17 genome fully covers the OriL sequence, and therefore, the OriL sequence was reinserted into the contigs after PCR verification. Internal repeat short (IRS), unique short (US), and terminal repeat short (TRS) regions were assembled into single contigs, which also showed some reduction in the length of several tandemly repeated sequences. The whole genome was built by joining contigs according to the order established by the alignment against strain 17, filling gaps with an Ns. Then, gaps were reduced using the SOAP*denovo* GapCloser tool (9), correcting 820 out of 7,453 Ns introduced to join contigs.

### Accession number(s).

The HSV-1 strain SC16 genome sequence (151,334 bp) has been deposited in GenBank under the accession no. KX946970.
